# Artificial Intelligence-Based Solution in Personalized Computer-Aided Arthroscopy of Shoulder Prostheses

**DOI:** 10.3390/jpm12010109

**Published:** 2022-01-14

**Authors:** Haseeb Sultan, Muhammad Owais, Jiho Choi, Tahir Mahmood, Adnan Haider, Nadeem Ullah, Kang Ryoung Park

**Affiliations:** Division of Electronics and Electrical Engineering, Dongguk University, 30 Pildong-ro 1-gil, Jung-gu, Seoul 04620, Korea; haseebsltn@gmail.com (H.S.); malikowais266@gmail.com (M.O.); choijh1027@dongguk.edu (J.C.); tahirmahmood.cs@gmail.com (T.M.); adnanhaider@dgu.ac.kr (A.H.); nadeemullahonline@gmail.com (N.U.)

**Keywords:** shoulder arthroplasty, implant classification, artificial intelligence, ensemble network, shoulder implant system

## Abstract

Background: Early recognition of prostheses before reoperation can reduce perioperative morbidity and mortality. Because of the intricacy of the shoulder biomechanics, accurate classification of implant models before surgery is fundamental for planning the correct medical procedure and setting apparatus for personalized medicine. Expert surgeons usually use X-ray images of prostheses to set the patient-specific apparatus. However, this subjective method is time-consuming and prone to errors. Method: As an alternative, artificial intelligence has played a vital role in orthopedic surgery and clinical decision-making for accurate prosthesis placement. In this study, three different deep learning-based frameworks are proposed to identify different types of shoulder implants in X-ray scans. We mainly propose an efficient ensemble network called the Inception Mobile Fully-Connected Convolutional Network (IMFC-Net), which is comprised of our two designed convolutional neural networks and a classifier. To evaluate the performance of the IMFC-Net and state-of-the-art models, experiments were performed with a public data set of 597 de-identified patients (597 shoulder implants). Moreover, to demonstrate the generalizability of IMFC-Net, experiments were performed with two augmentation techniques and without augmentation, in which our model ranked first, with a considerable difference from the comparison models. A gradient-weighted class activation map technique was also used to find distinct implant characteristics needed for IMFC-Net classification decisions. Results: The results confirmed that the proposed IMFC-Net model yielded an average accuracy of 89.09%, a precision rate of 89.54%, a recall rate of 86.57%, and an F1.score of 87.94%, which were higher than those of the comparison models. Conclusion: The proposed model is efficient and can minimize the revision complexities of implants.

## 1. Introduction

The anatomy and biomechanics of the human shoulder, comprising different joints, are the most complicated parts of the human body [1]. Retroversion, ranging from 0 to 55° in the shoulder, varies across persons and between the left and right sides of the same person [2]. Medical practitioners diagnose the pain and examine injuries to the shoulders using a physical examination or imaging tests on the joints [3]. Hemiarthroplasty, total shoulder arthroplasty (TSA), and reverse total shoulder arthroplasty (RTSA) are surgical procedures to treat shoulder arthritis and relieve severe pain [4,5]. In these treatments, a prosthesis is used to reconstruct the impaired shoulder, restore its movement, and relieve pain. A linear regression analysis performed on the National Inpatient Sample (NIS) database anticipates that the volume of TSA and RTSA will increase to 91.9% by the year 2025 [6]. 

A shoulder arthroplasty needs to be revised over time for different reasons, such as a severe fracture, deep infection in the wound, loosened or dislocated implant, or failure of the previous surgery [7,8]. In 2017, the cost to the US healthcare system for the revision of 10,290 shoulder arthroplasty patients was anticipated to be 205 millon dollars, which has been increasing annually [9]. Orthopedic prostheses are made of highly developed biomaterials. Owing to their intricate designs, they vary by model and manufacturer and have an effect on the apparatus alignment [10]. Identifying and properly seating prostheses is a crucial surgical step that helps avoid common complications, such as bone and blood losses. Therefore, selecting and identifying the correct prosthesis model for a particular patient is necessary for personalized medicine. 

Because of the exponential prevalence of shoulder arthroplasty, a greater demand exists for well-qualified orthopedic surgeons who specialize in revision shoulder arthroplasty [9]. Indeed, surgeons have limited experience with a small number of implants to enhance their technological expertise [11]. They examine the X-ray scans of implants to recognize them. However, this manual examination is time-consuming and surgeon-dependent. Each year, surgeons and medical staff spend 41 h on identifying and revising implants for patients [12]. Inaccurately planted implants increase the chances of component failure, dislocation, and the need for revision surgery [13]. Certain old implant models have been discontinued, whereas manufacturers continue to develop new models that differ from the earlier versions. Replacement and repair of obsolete prosthesis models necessitate the use of particular methods and equipment. Therefore, it is fundamental to identify an appropriate model. Furthermore, doctors’ preferences for prostheses vary over time. In some cases, surgeons and patients may be unaware of the implant’s manufacturer and model when the initial medical treatment is conducted outside the county and patients are unable to access their medical records. Patients who switch to hospitals for revision encounter a greater risk of complications [14]. Because of the inconsistencies in the documentation and global restrictions, medical practitioners are unable to identify the implant model and its manufacturer [14]. Another reason is that the initial surgery may have been performed several years before the subsequent surgery, and the medical information of the patient might have been misplaced or imprecise. In such circumstances, medical specialists visually compare the imaging tests and an implant atlas [15] to identify a prosthesis. This task is laborious, time-consuming, and dependent on the surgeon’s skills. Incorrect identification of prostheses may have serious consequences. Failure to identify a failed implant results in the replacement of more components, loss of more blood, destruction of more bone, and, thus, a longer recovery period. Moreover, elderly patients are at a high risk of surgical complications and are less likely to benefit from a revision with a new implant [16]. In such cases, it is difficult for surgeons to identify implant models owing to their limited experience and lack of documentation, which causes a lack of knowledge about these models. Indeed, a minor error may have severe consequences. Therefore, it is necessary to design an efficient automated framework to address these problems. 

Despite considerable advances in pattern recognition and deep learning (DL) in the medical field, studies on categorizing shoulder implants have been relatively limited. In this study, we propose an automated framework based on DL for identifying shoulder prostheses to assist medical practitioners in preoperative planning to avoid surgical complexity and reduce medical costs. The main contributions of this study are as follows.

To classify the shoulder implant X-ray scans of different patients, we removed the average pooling layer and inculcated a convolutional pooling (CP) block in Inception-V3 and designed an inception fully-connected convolutional network (IFC-Net). Our IFC-Net outperforms the existing methods and achieves higher accuracy. The CP block enables the network to extract the optimum features that are lost by the average pooling layer. 

We inculcated a CP block in MobileNet-V2 and designed a mobile fully-connected convolutional network (MFC-Net). 

We further improved the results by designing an inception mobile fully-connected convolutional network (IMFC-Net), which is an ensemble of our IFC-Net, MFC-Net, and a joint multilayer perceptron (JMLP) network. Our IMFC-Net achieves higher accuracy than that of our IFC-Net and all state-of-the-art methods. 

Our model is publicly available [17], which allows other researchers to make fair comparisons.

The remainder of this paper is organized as follows: The proposed classification framework is described in Section 2. The experimental setup and results are presented in Section 3. Finally, Section 4 and Section 5 present discussions and draw conclusions, respectively.

## 2. Materials and Methods

### 2.1. Dataset

**Figure 1:**
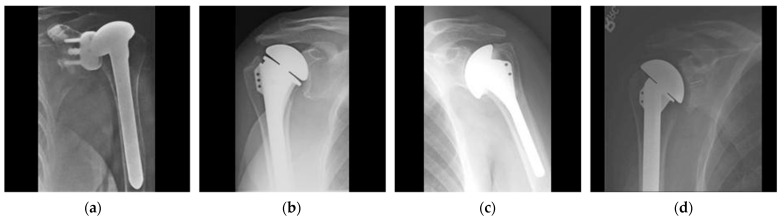
Example images of the four manufacturers: (**a**) Cofield, (**b**) Depuy, (**c**) Tornier, and (**d**) Zimmer.

The experimental results based on ten-fold cross-validation were evaluated using a publicly available shoulder implant dataset [18,19]. The dataset contained 597 shoulder implant X-ray scans that were categorized into four classes, considering the manufacturers. The dataset was collected at the Biomedical Image and Data Analysis Lab (BIDAL), San Francisco. X-ray scans were captured at different angles and exhibited certain patterns of holes and fins. The example scans of each manufacturer are shown in Figure 1. The four manufacturers, Cofield, Depuy, Tornier, and Zimmer, had 83, 294, 71, and 149 X-ray scans, respectively. Figure S1 shows the high intra-class and low inter-class variations of the manufacturers. Imbalanced distribution of the dataset and the high intra-class and low inter-class variations make classification a challenging task. All convolutional neural networks (CNNs) were trained, validated, and tested using different patient datasets and ten-fold cross-validation. We constructed the ten-fold cross-validation of 597 implant models by splitting 90% of the data into training sets, 2% of the data into validation sets, and the remaining 8% of the data into testing sets. Table S1 shows the RIA [20] training, validation, and testing data for ten-fold cross-validation. 

### 2.2. Overall Workflow

**Figure 2:**
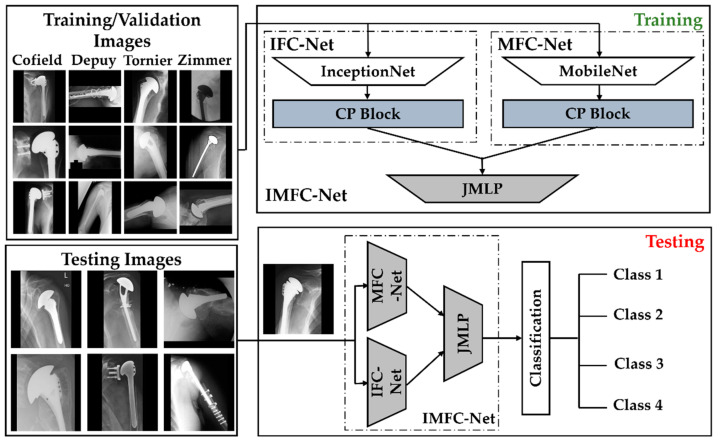
Flow diagram of the proposed inception mobile fully-connected convolutional network (IMFC-Net), which is an ensemble of inception fully-connected convolutional network (IFC-Net), mobile fully-connected convolutional network (MFC-Net), and a joint multilayer perceptron (JMLP) network.

An overall flow diagram of the proposed method is presented in Figure 2. First, the images were input to the network, and augmentation was performed using the rotational invariant augmentation (RIA) [20] technique during training. However, augmentation was not performed on the validation and testing datasets. Subsequently, IFC-Net and MFC-Net were trained and validated. The final classification of the test image was made in the testing phase based on the IMFC-Net output. The proposed framework was designed to propose a network to minimize the number of parameters, achieve high performance, and classify a shoulder implant test image accurately.

The proposed IMFC-Net is based on the ensemble connectivity of our designed IFC-Net and MFC-Net, followed by JMLP, as shown in Figure S2. Previous studies [20,21] have demonstrated that an ensemble strategy based on the joint usage of multiple features is more likely to attain the optimum performance for medical image classifications. Although the experimental results showed that our proposed IFC-Net outperformed all previous state-of-the-art methods, we further improved the performance by designing IMFC-Net. The proposed IMFC-Net outperformed IFC-Net and MFC-Net. Moreover, the proposed IMFC-Net encompassed fewer parameters than the previous ensemble model for the problem under investigation. The proposed network (IMFC-Net) obtains the input image and extracts the optimum features (***f_I_*** and ***f_M_***) using two CNNs (IFC-Net and MFC-Net). The detailed layer-wise architectures of IFC-Net and MFC-Net are shown in Tables S2 and S3 respectively. Finally, the features are concatenated (***f_IM_***) using a third network (JMLP) for the final classification. JMLP provides an extra performance boost over the simple ensemble of IFC-Net and MFC-Net. The detailed layer-wise architecture of JMLP is shown in Table S4. 

#### 2.2.1. Model Design

In general, the deeper the CNN, the more likely it is to achieve high performance [22,23,24]. In ensemble learning, different models are combined into a single deep, high-quality classifier to improve the prediction performance. An ensemble of deep CNNs improves the accuracy through a trade-off between the size and speed of the network [25]. We designed an efficient ensemble network called IMFC-Net, which is comprised of IFC-Net, MFC-Net, and JMLP. To achieve maximum performance gain, we designed IFC-Net based on Inception-V3 [23], which was pre-trained on the ImageNet dataset [26]. To keep the size of the ensemble network to a minimum, we designed MFC-Net, based on MobileNet-V2 [27], which was pre-trained on the ImageNet dataset [26]. In Inception-V3, convolutional (Conv) layers are efficiently scaled up by maintaining a modest computational cost. This is made possible by the appropriate use of parallel structures of inception modules with dimensional reduction. MobileNet-V2 was selected as the base net for MFC-Net because of its lower memory consumption, smaller size, smaller number of parameters, and real-time performance in real-world applications. The high efficiency of MobileNet-V2 is owed to the reasonable use of depthwise separable convolutions and inverted residual blocks in its architecture. 

We designed IFC-Net and MFC-Net by introducing a novel CP block in Inception-V3 and MobileNet-V2, respectively. The detailed layer-wise structure of IFC-Net and MFC-Net is presented in Tables S2 and S3, respectively. In detail, the architecture of our IFC-Net consisted of different inception modules that were grouped into different blocks named ‘Block A’, ‘Block B’, ‘Block C’, ‘Block D’, ‘Block E’, and ‘Block CP’. A complete description of the first five blocks can be found in [23]. The architecture of MFC-Net was comprised of different Conv layers and depthwise-Conv (DW-Conv) layers with a different number of filters and kernel sizes, which were grouped into different blocks named ‘Block A’, ‘Block B’, and ‘Block CP’. A detailed description of Blocks A and B was presented in [27]. Here, we discuss the architecture and importance of our novel CP block of IFC-Net and MFC-Net (Block CP in Tables S2 and S3).

A. CP block of IFC-Net

Our novel CP block of IFC-Net comprises different layers labeled ‘IFC-Conv’, ‘BN’, ‘ReLU’, ‘IFC-FC’, and ‘ReLU’, as shown in Figure S3a. In the Inception-V3 architecture, after ‘Block E’, the average pooling layer of the filter size 8×8 pixel resolution is used to reduce the dimension, which causes the loss of useful features. We need to preserve the optimum features as our dataset comprises high intra-class and low inter-class images, as shown in Figure S1. Therefore, we removed the average pooling layer and introduced the ‘CP block’ to avoid degradation of the classification performance. 

The experimental results demonstrated the usefulness of ‘Block CP’ over average pooling. The proposed ‘Block CP’ (CP block in Figure S2) of IFC-Net held the input feature map with a pixel resolution of 8×8×2048 and processed it as follows. The ‘IFC-Conv’ layer in the CP block exploited the optimum features by using 50 filters with a kernel size of 8×8 and stacking the activation maps of all filters to an activation map with a pixel resolution of 8×8×50. Subsequently, high-level representations of implant images were exploited using the ‘IFC-FC’ layer of the CP block. This layer combines all features of the activation map 8×8×50  pixel resolution into a one-dimensional (1D) feature vector (***f_I_***) with a resolution of 1 ×1×64 pixels. This layer consists of 64 nodes connected to all activations of the previous activation map. Mathematically, ***f_I_*** can be obtained by multiplying a weight matrix (***W***) by the flattening tensor (***F_I_***) of the previous layer of dimensions *hi*, *w_i_*, and *c_i_* as $f_{I}=W\times F_{I}+b$, where b is a bias vector and *hi*, *w_i_*, and *c_i_* are the height, width, and channel of ***F_I_***, respectively. A batch normalization (BN) layer and a rectified linear unit (ReLU) layer were inserted in the CP block for re-parametrizing and achieving an efficient computation. The negative values of $f_{I}$ were suppressed by applying an activation function through the ‘ReLU’ layer. Statistically, it is defined as $f\left(f_{I}\right)=max\left(0,f_{I}\right).$ The experimental results showed that IFC-Net outperformed all state-of-the-art methods. Moreover, IFC-Net is efficient, with 31.72% fewer parameters than that of the presented method in [20] (i.e., 41.7 M [20] > 28.4 M (IFC-Net)) and 2.14% higher accuracy than that achieved in [20]. We designed IMFC-Net based on IFC-Net to enhance the classification performance. To this end, the optimized features from ‘Block CP’ of IFC-Net were extracted and concatenated with the optimized features of MFC-Net through JMLP.

B. CP block of MFC-Net

We modified MobileNet-V2 by inserting the CP block, which resulted in increased classification performance. The experimental results proved that the average pooling layer diminished the optimum features of the implants, while the CP block empowered them. This novel block comprises different layers labeled ‘MFC-Conv’, ‘BN, ‘ReLU’, ‘MFC-FC’, and ‘ReLU’, as shown in Figure S3b. The proposed ‘Block CP’ (CP block in Figure S2) of MFC-Net held an input feature map with a pixel resolution of 7×7×1280 and was processed. The ‘MFC-Conv’ layer in the CP block exploited the optimum features by using 50 filters with a kernel size of 7×7 pixel resolution and stacking activation maps of all filters to an activation map with a resolution of 7×7×50 pixels. Subsequently, high-level representations of implant images were exploited using the ‘MFC-FC’ layer of the CP block. This layer combines all features of the activation map 7×7×50 pixel resolution into a 1D feature vector (***f_M_***) with a resolution of 1 ×1×64 pixels. This layer consists of 64 nodes connected to all activations of the previous activation map. Mathematically, ***f_M_*** can be obtained by multiplying a weight matrix (***W***) by the flattening tensor (***F_M_***) of the previous layer of dimensions *h_m_*, *w_m_*, and *c_m_* as $f_{M}=W\times F_{M}+b$, where b is a bias vector and *h_m_*, *w_m_*, and *c_m_* are the height, width, and channel of ***F_M_***, respectively. The BN and ReLU layers were inserted into the CP block for re-parametrizing and achieving an efficient computation. The negative values of $f_{M}$ were suppressed by applying an activation function through the ‘ReLU’ layer. Mathematically, it is defined as $f\left(f_{M}\right)=max\left(0,f_{M}\right).$ The experimental results proved that our proposed MFC-Net achieved 3.38% higher accuracy than that of the presented method in [19]. We designed IMFC-Net based on MFC-Net to enhance the classification performance. To this end, the optimized features from the ‘CP Block’ of MFC-Net were extracted and concatenated with the optimized features of IFC-Net through JMLP.

C. Feature Concatenation and Final Classification by JMLP

The high-level features ($f_{I}\;\mathrm{and}\;f_{M}$) extracted from the respective CP blocks of IFC-Net and MFC-Net are concatenated as $f_{IM}$ along the depth direction through JMLP, as shown in Figure S2. A detailed layer-wise architecture of JMLP is provided in Table S4. Two input feature vectors, $f_{I}\;\mathrm{and}\;f_{M}$, with a resolution of 1 ×1×64 pixels, were provided to the first layer (Concat) of JMLP for depth concatenation. The ‘Concat’ layer concatenated $f_{I}\;\mathrm{and}\;f_{M}$ and generated an optimized deep feature vector $f_{IM}$ with a resolution of 1 ×1×128 pixels. Moreover, the JMLP network was filled with three FC layers labeled as ‘FC-1’, ‘FC-2’, and ‘FC-3’, one softmax layer labeled as ‘Softmax’, and one classification layer labeled as ‘Classification’, as presented in Table S4. The first two fully connected (FC) layers (FC-1 and FC-2) of JMLP consisted of 64 nodes each, and the last ‘FC-3’ layer consisted of four nodes equal to the number of classes. The ‘FC-1’ and ‘FC-2’ layers were responsible for manipulating the optimum features in $f_{IM}$ using learnable parameters ***W*** and ***b***, where W and b  represent the trainable weights and the bias vector, respectively. Finally, the final ‘FC-3’ layer exploited the large pattern of the output feature vector of the previous layers (‘FC-1’ and ‘FC-2’) and generated a smaller 1D feature vector (f) with a resolution of 1 ×1×4 pixels. Mathematically, it is expressed as $f=W\times f_{IM}+b,$ where $f_{IM}$ is the 1×1×64 output feature vector of the ‘FC-2’ layer and f = [***f_im_***|***_im_*** *_=_*_1,2,3,4_]. Subsequently, a SoftMax function [28] was applied using the ‘Softmax’ layer to obtain $f^{IM}$, which is the probability distribution of f. The SoftMax function is expressed as $f^{IM}=e^{f}/\overset{4}{\underset{im=1}{\sum }}e^{f_{im}},$ where $e^{f}$ is the exponential of f. Finally, the ‘Classification’ layer assigned one of the four class labels to each probability value of $f^{IM}$. The experimental results proved that our proposed IMFC-Net achieved a higher accuracy than that of IFC-Net and all state-of-the-art methods. 

## 3. Results

This section describes the experimental setup and obtained results of the proposed methods in comparison to those of the state-of-the-art methods. Moreover, the ablation studies of IFC-Net, MFC-Net, and IMFC-Net are analyzed and discussed.

### 3.1. Experimental Setup and Network Training

We implemented all our proposed models on a Windows 10 operating system using the DL toolbox of MATLAB R2020b (MathWorks, Inc., Natick, MA, USA) [29]. The system was equipped with an NVIDIA GeForce GPU (GTX-1070), 16 GB of RAM, and an Intel (R) Core (TM) i5-2320 CPU. We used the cross-entropy (CE) loss [28] and an optimization algorithm of stochastic gradient descent (SGD) [30] for our proposed scheme. The CE loss was obtained by assigning the final class label to a test image and calculating the negative loss of $f^{IM}$. Mathematically, it is expressed as $CE\;loss=-\overset{4}{\underset{im=1}{\sum }}t_{im}log(f^{IM})$, where $t_{im}=1$ for a true label class. During training, SGD minimizes the objective function ***j***(***θ***) by updating the parameter ***θ*** for each representative implant of a class in the reverse direction of the gradient of ***j***(***θ***). Moreover, $f^{IM}$ denotes a training example, its label is represented by $y^{IM}$, and θ is updated as $\;\theta =\theta -\eta \times \nabla j\left(\theta ;f^{IM};y^{IM}\right).$ For the optimal convergence benchmark, the learning rate η was set to 0.001 to follow the slope set by ***j***(***θ***) and reach a minimum. The other training hyperparameters were used as default values provided by MATLAB R2020b, such as momentum factor = 0.9, L2-regularization = 0.0001, and learning rate drop factor = 0.1. However, the number of epochs varied for training different networks. For a sequential training, the number of epochs was 13, 13, and 4 for IFC-Net, MFC-Net, and IMFC-Net, respectively, with a mini-batch size of 10. The networks, IFC-Net and MFC-Net, were trained independently on 90% of the given dataset (RIA-Training data in Table S1) based on ten-fold cross-validation. During the training of the proposed IMFC-Net, the learnable weights of the independently trained networks (IFC-Net and MFC-Net) were frozen, and their parameters were extracted and concatenated through JMLP for a final prediction of 8% of the given dataset (Testing data in Table S1). For fair training and testing, the ten-fold cross-validation was the same for training and testing of independently trained networks (IFC-Net and MFC-Net) and the ensemble network (IMFC-Net).

Figure S4 shows the training-validation accuracy and loss graphs for all three proposed networks. Figure S4 demonstrates that the training accuracies and losses were 100 and 0%, respectively. Therefore, all networks were sufficiently trained and converged satisfactorily. It is interesting to note that the number of training epochs for both Figure S4c (IMFC-Net sequential) and Figure S4d (IMFC-Net end-to-end) was the same, but their training elapsed times were different. The average calculated training elapsed time for Figure S4c (IMFC-Net sequential) was 1.6 h, whereas, for Figure S4d (IMFC-Net end-to-end), it was 2.5 h. For sequential training of IMFC-Net, the initially trained parameters (***p_I_*** and ***p_M_***) of IFC-Net and MFC-Net were obtained to perform transfer learning to the target domain using JMLP. The weights of IFC-Net and MFC-Net were frozen, and JMLP was trained from scratch with the initial parameters ***p_I_*** and ***p_M_***. Therefore, the training time of IMFC-Net sequential, which showed more acceptable testing results, was less than that of IMFC-Net end-to-end. For a small dataset, training from scratch causes overfitting and lag in performance gain. To address overfitting and generalization issues, we considered the data of different patients in the training, validation, and testing phases. The validation graphs of accuracies and losses in Figure S4 present the optimal convergence of all networks without overfitting the training data. 

After training and validation, the proposed models, including state-of-the-art models, were evaluated in terms of accuracy (ACC), F1.score (F1), average precision (AP), and average recall (AR). Moreover, the assessment matrices are defined as ACC=(TP+TN)/(TP+TN+FP+FN), AP=TP/(TP+FP), AR=TP/(TP+FN), and F1=(2×AP×AR)/(AP+AR), where *TP*, *TN*, *FP*, and *FN* represent the true positive, true negative, false positive, and false negative, respectively. In particular, *TP* and *TN* are the correctly predicted positive and negative cases by our proposed network for all four manufacturers, whereas *FP* and *FN* are the incorrectly predicted positive and negative cases by our network for all four manufacturers, respectively.

### 3.2. Our Results (Ablation Studies)

We considered the ablation studies in two parts: (1) ablation studies of IFC-Net and MFC-Net concerning the CP block, and (2) the ablation study of IMFC-Net. In the first ablation study, we demonstrated the significance of the CP block for both IFC-Net and MFC-Net. Table 1 shows the significant differences between the results obtained with and without the CP block. This block extracts the optimum features of the high inter-class variability shoulder implant dataset. In the modified Inception-V3, the difference between the results obtained with and without using the CP block is 1.45% for ACC, 1.41% for AP, 2.56% for AR, and 2.03% for F1. Moreover, in the modified MobileNet, the difference between the results obtained with and without using the CP block is 0.64% for ACC, 1.88% for AP, 0.74% for AR, and 1.23% for F1. We gradually improved the design of the CP block by investigating the effect of its learnable-weight layers on IFC-Net. We removed the average pooling layer of Inception-V3 and added a Conv layer. Consequently, ACC, AP, AR, and F1 were incremented by 1.04%, 0.93%, 2.46%, and 1.74%, respectively. Subsequently, we added an FC layer of 64 nodes, which increased ACC, AP, AR, and F1 by 0.41%, 0.48%, 0.1%, and 0.29%, respectively. 

In the ablation study of the proposed IMFC-Net, we considered three cases: (1) the role of each submodule of IMFC-Net, (2) sequential training of IMFC-Net vs. end-to-end training of IMFC-Net, and (3) a comparison between the performance of IMFC-Net and that of the base models [23,27]. First, we compared the testing results obtained using IMFC-Net with its submodules, as shown in Table 2. The ensemble of different DL models strengthens each other and exhibits a higher performance gain than that of the stand-alone model. The experimental results in Table 2 confirmed the significance of each subnetwork in the proposed ensemble network. The performance gain of the proposed network, which is the ensemble of IFC-Net and MFC-Net, is significantly higher than that of its stand-alone subnetworks. The significant difference between the performance gain of the proposed network and its submodule (IFC-Net), which is also the second-best network, is 1.87% for ACC, 1.88% for AP, 2.25% for AR, and 2.06% for F1, as presented in Table 2. However, the other submodule (MFC-Net) of the proposed ensemble network is not the third-best network, but it still boosts the performance gain of the proposed network. We used MFC-Net in our proposed ensemble model to minimize the number of parameters and maximize the performance gain, as discussed in Section 2.2.1.

In the second ablation study of IMFC-Net, we compared the performance of sequential training with that of the end-to-end training of IMFC-Net. Table 3 demonstrates the superiority of the sequentially trained IMFC-Net over the end-to-end-trained IMFC-Net by presenting a significant difference in ACC, AP, AR, and F1, which are 2.53%, 4.01%, 2.5%, and 3.24%, respectively. Our ensemble model comprises one high-capacity model (IFC-Net) with 28.4 M parameters and one low-capacity model (MFC-Net) with 5.5 M parameters. Owing to the small size of the dataset, the results of the end-to-end-trained proposed network underwent the dominant effect of the high-capacity model. Therefore, the results of the proposed end-to-end network (Table 3) exhibit a small difference from those of IFC-Net (Table 2) as compared to MFC-Net (Table 2). In the sequential training of the proposed network, weights of the independently trained subnetworks were frozen. However, JMLP was trained from scratch using the parameters ***p_I_*** and ***p_M_***. Therefore, this training method is fast and robust for small datasets.

**Figure 3:**
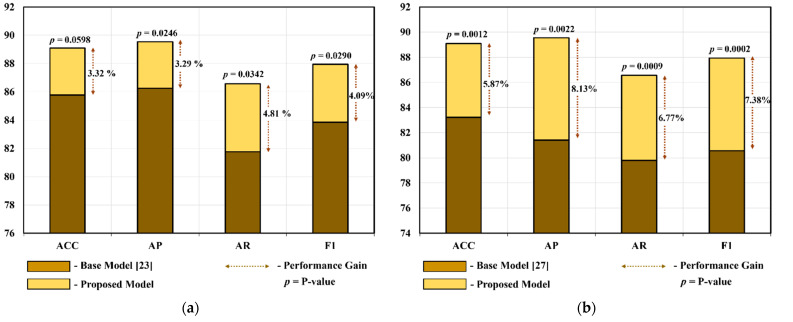
Significant performance gain of the proposed model using the t-test analysis in terms of accuracy (ACC), average precision (AP), average recall (AR), and F1.score (F1): (**a**) difference between the base model presented in [23] and the proposed model (*p*-values), as well as the performance gain obtained using the proposed model compared to that of the base model [23], and (**b**) difference between the base model presented in [27] and the proposed model (*p*-values), along with the performance gain obtained using the proposed model compared to that of the base model [27].

In the third case of the ablation study of IMFC-Net, we performed a statistical analysis (*t*-test) that revealed a substantial inconsistency between our proposed model and the comparison models. The *t*-test analysis is performed to robustly prove that a significant statistical difference exists [31]. We carried out a *t*-test analysis on the values of ACC, AP, AR, and F1 of all ten-folds of the base and proposed models. Figure 3a shows that we performed a *t*-test analysis for the base model presented in [23] and obtained *p*-values of 0.0599 for ACC, 0.0246 for AP, 0.0343 for AR, and 0.029 for F1. The average calculated *p*-value was 0.037, which is less than 0.05, indicating that our model significantly differs from the model presented in [23], with a confidence level of 95%. Similarly, we performed a *t*-test analysis for the base model presented in [27] and obtained *p*-values of 0.0012 for ACC, 0.0002 for AP, 0.001 for AR, and 0.0002 for F1, as shown in Figure 3b. The average calculated *p*-value was 0.0007, which is less than 0.01, and shows that our model considerably differs from the model presented in [27], with a confidence level of 99%. In addition, Figure 3 shows the significant quantitative performance gain of our proposed model over the base models [23,27]. As shown in Figure 3a, the average performance gain values of IMFC-Net over the base model [23] in terms of ACC, AP, AR, and F1 are 3.32%, 3.29%, 4.81%, and 4.09%, respectively. Similarly, Figure 3b shows that the average performance gain values of IMFC-Net over the base model presented in [27] in terms of ACC, AP, AR, and F1 are 5.87%, 8.13%, 6.77%, and 7.38%, respectively. 

**Figure 4:**
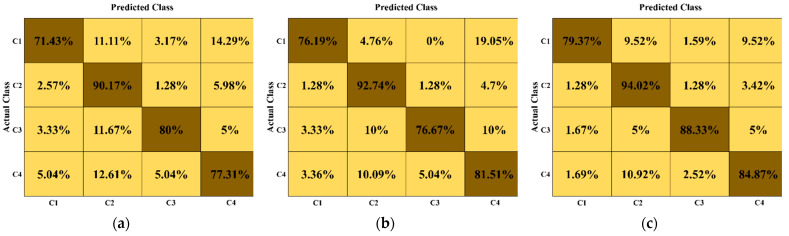
A comparison between the performance of the proposed IMFC-Net with that of the base models in terms of confusion matrices (unit: %): (**a**) the confusion matrix of the base model [27], (**b**) the confusion matrix of the base model [23], and (**c**) the confusion matrix of the proposed model. (C1: Cofield manufacturer, C2: Depuy manufacturer, C3: Tornier manufacturer, and C4: Zimmer manufacturer).

Moreover, we demonstrated the considerable performance of our model for each class in terms of the confusion matrix. The matrices in Figure 4 particularly characterize the anticipated number of TP, TN, FP, and FN data samples for the base models [23,27] and IMFC-Net. The diagonal elements of these matrices indicate the AR for each class. Our proposed model outperformed the base models with a considerable difference for each class, as shown in Figure 4. The AR value in Figure 4b (base model [23]) for all classes is greater than that of Figure 4a (base model [27]), except for C3. The underlying reason is discussed in Section 4, with a visual explanation of IFC-Net and MFC-Net. Figure 4c (the proposed model) shows that the AR value for each class is higher than 80%, except for C1. The underlying reason is discussed in Section 4 by analyzing C1 for the FN cases.

### 3.3. Comparisons

We thoroughly compared the proposed model with different state-of-the-art models with and without augmentation. The comparison models included VGG-16 [22], VGG-19 [22], DarkNet-53 [32], NASNet [33], ResNet-18 [24], ResNet-50 [24], ResNet-101 [24], DenseNet-201 [34], Inception-V3 [23], MobileNet-V2 [27], and DRE-Net [20], and the comparison was performed by augmenting the dataset using the RIA and random in-plane translation and rotation augmentation (online augmentation). In addition, we compared the performance of the proposed model with that of the comparison models [20,22,23,24,27,32,33,34] without augmentation. Table 4, Table 5 and Table 6 present the quantitative performance evaluation results using the RIA, online augmentation, and without augmentation, respectively. The experimental results revealed that our model outperformed the comparison models in all three cases. The existing methods for the classification of the shoulder implants have not used validation datasets. Therefore, we aimed to include a validation dataset to perform a fair comparison and validate all comparison models using the transfer learning with our dataset. To this end, all experimental results in Table 4 were evaluated using RIA, which proved that our model outperformed all the comparison models. Table 4 shows that the second-best network is Inception-V3 [23], with an ACC and AP of 3.32% and 3.29% less than that of our model, respectively. Furthermore, Table 4 demonstrates that DRE-Net [20] is the second-best model, with an AR and F1 of 2.96% and 3.79% less than that of our model, respectively. Furthermore, our IMFC-Net outperforms the model presented in [20] in terms of performance gain, where it has 18.4% fewer parameters than that of the model presented in [20] (i.e., 41.7 M [20] > 34 M (Proposed)). 

All experimental results in Table 5 were evaluated using the online augmentation for a fair comparison, and our proposed model outperformed all the comparison models. Table 5 shows that the second-best network is DenseNet-201 [34] with an ACC, AR, and F1 of 3.37%, 5.06%, and 4.3% less than that of our model, respectively. As for AP, ResNet-101 [24] is the second-best network, with an AP of 3.09% less than that of our proposed model. All experimental results in Table 6 were evaluated without augmenting the training data for a fair comparison, and our model outperformed all the comparison models. Table 6 shows that the second-best network is ResNet-50 [24], with an ACC, AP, AR, and F1 of 11.76%, 11.27%, 14.61%, and 13.09% less than that of our model. 

The experimental results proved that the second-best models using RIA are Inception-V3 [23] and DRE-Net [20], the second-best models using the online augmentation are DenseNet-201 [34] and ResNet-101 [24], and the second-best model without augmentation is ResNet-50 [24]. Therefore, different augmentation techniques have different effects on various CNNs. However, in all three cases, our model (IMFC-Net) ranked first, with a considerable difference from the second-best models, demonstrating its generalizability.

**Table 5:** Comparative performance analysis of the average results of ten-fold cross-validation between the state-of-the-art and proposed models using random in-plane translation and rotation augmentation (online augmentation) for the shoulder implant dataset. (ACC: accuracy, AP: average precision, AR: average recall, F1: F1.score, Std: standard deviation, unit: %).

Model	ACC ± Std	AP ± Std	AR ± Std	F1 ± Std
VGG-16 [19,20,22]	72.97 ± 7.9	73.76 ± 8.39	69.16 ± 8.79	71.21 ± 7.7
VGG-19 [19,20,22]	67 ± 8.77	69.48 ± 10.15	61.42 ± 10.04	64.93 ± 9.8
DarkNet-53 [32]	51.66 ± 7.68	38.64 ± 8.55	39.44 ± 7.64	38.99 ± 7.98
NASNet [19,20,33]	70.8 ± 4.69	68.56 ± 6.59	62.78 ± 9.04	65.34 ± 6.95
ResNet-18 [20,24,35]	71.41 ± 5.81	69.9 ± 10.06	64.94 ± 7.41	67.03 ± 7.48
ResNet-50 [19,20,24]	80.26 ± 4.17	79.13 ± 5.45	74.93 ± 4.5	76.93 ± 4.52
ResNet-101 [24]	79.39 ± 6.44	79.27 ± 7.98	75.37 ± 8.37	77.14 ± 7.42
DenseNet-201 [19,20,34]	80.45 ± 4.77	78.68 ± 6.24	76.02 ± 5.9	77.26 ± 5.52
Inception-V3 [23]	76.23 ± 4.27	74.5 ± 6.01	68.48 ± 5.53	71.32 ± 5.46
MobileNet-V2 [27]	71.02 ± 6.56	67.51 ± 7.51	64.09 ± 8.8	65.7 ± 7.96
DRE-Net [20]	77.11 ± 5.35	78.14 ± 7.09	72.83 ± 5.33	75.21 ± 4.86
Proposed (IMFC-Net)	83.82 ± 3.12	82.36 ± 4.90	81.08 ± 5.27	81.56 ± 3.55

**Table 6:** Comparative performance analysis of the average results of ten-fold cross-validation between the state-of-the-art and proposed models without using augmentation for the shoulder implant dataset [18,19]. (ACC: accuracy, AP: average precision, AR: average recall, F1: F1.score, Std: standard deviation, unit: %).

Model	ACC ± Std	AP ± Std	AR ± Std	F1 ± Std
VGG-16 [19,20,22]	64.48 ± 6.57	61.84 ± 8.7	59.54 ± 8.57	60.63 ± 8.5
VGG-19 [19,20,22]	66.77 ± 6.34	65.38 ± 8.79	61.57 ± 8.51	63.26 ± 7.87
DarkNet-53 [32]	48.12 ± 7.8	39.3 ± 7.69	36.87 ± 7.17	38.02 ± 7.37
NASNet [19,20,33]	58.18 ± 6.65	54.52 ± 8.66	49.06 ± 7.57	51.49 ± 7.61
ResNet-18 [20,24,35]	62.53 ± 8.02	59.72 ± 10.32	54.7 ± 10.34	56.97 ± 9.89
ResNet-50 [19,20,24]	70.17 ± 5.87	68.95 ± 6.78	63.37 ± 7.856	65.93 ± 6.76
ResNet-101 [24]	66.19 ± 6.31	65.89 ± 8.35	59.5 ± 8.04	62.42 ± 7.64
DenseNet-201 [19,20,34]	62.18 ± 6.55	53.95 ± 9.48	52.31 ± 8.08	53.04 ± 8.52
Inception-V3 [23]	69.15 ± 5.29	67.37 ± 7.49	62.9 ± 6.98	65 ± 6.86
MobileNet-V2 [27]	64.06 ± 7.23	60.78 ± 10.95	57.35 ± 9.13	58.95 ± 9.78
DRE-Net [20]	56.27 ± 5.39	50.1 ± 7.52	47.95 ± 7.39	48.96 ± 7.29
Proposed (IMFC-Net)	81.93 ± 3.3	80.22 ± 4.7	77.98 ± 6.23	79.02 ± 4.98

## 4. Discussion

After the successful application of the DL models in object detection, classification, and localization, various DL algorithms have been successfully used to design classification [36,37] and segmentation frameworks [38,39,40] to diagnose different diseases. However, the use and potential advantages of DL-based models in arthroplasty are limited. Tables S5 and S6 contain a comprehensive literature review on the classification of various types of implants in radiographs. In the literature, handcrafted feature-based methods, as well as different DL-based methods, have been described for the classification of different types of dental implants [41,42,43,44,45,46]. Similarly, artificial intelligence (AI)-based systems were designed to identify hip and knee implants in radiographs [47,48,49,50,51,52,53,54,55]. However, a few DL-based studies have been conducted to recognize shoulder implants based on manufacturers. A DL system was proposed in [35] for the binary classification of shoulder implants. TSA and RTSA were classified using a pre-trained residual network based on transfer learning techniques. Five types of TSA implant models were classified using a separate classifier for each model. An implant dataset was collected from online archives. Therefore, the authenticity of the label was questioned. In [19], the first DL-based study was presented for the classification of shoulder prostheses supplied by four different manufacturers. In addition, the non-DL and DL algorithms were compared, in addition to a comparison between the pre-trained and non-pre-trained DL models. Ten-fold trials were performed using various pre-trained CNNs, which yielded a maximum accuracy of 80%. However, the validation dataset was not used, and the experiments were limited to a closed-world scenario. A DL-based ensemble network was proposed for the robust classification of various shoulder prostheses [20]. The proposed network in [20] outperformed the method presented in [19] by achieving an accuracy of 85.92%. However, their ensemble model was replete with many parameters, and the state-of-the-art methods were not validated using a validation dataset. For a fair comparison, we used a validation dataset to validate all state-of-the-art methods, including our proposed networks. 

**Figure 5:**
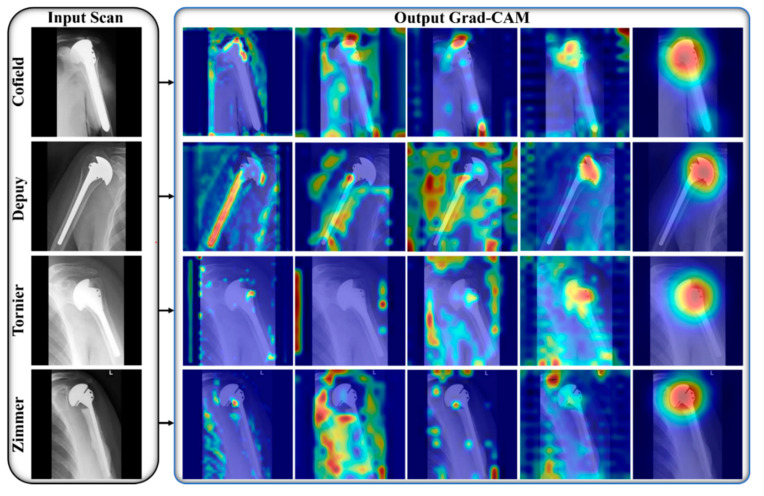
Visual explanation maps of IFC-Net for all manufacturers. The 1st to 5th Grad-CAM images are, respectively, obtained from Block A, Block B, Block C, Block D, and Block E in Table S2.

**Figure 6:**
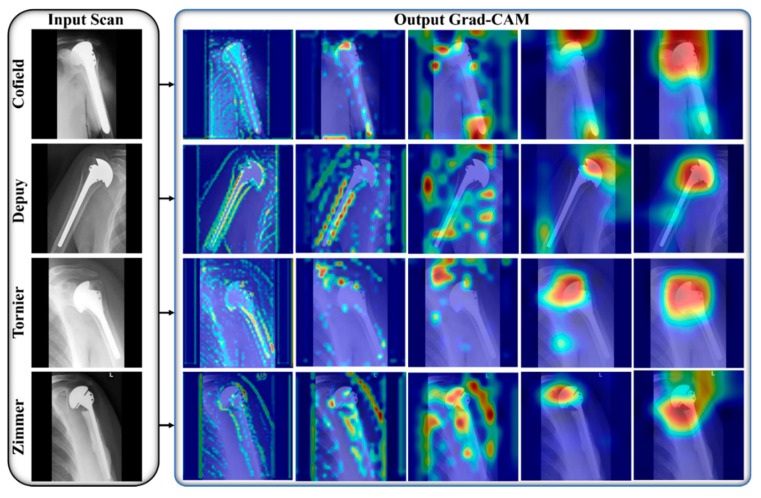
Visual explanation maps of MFC-Net for all manufacturers. The 1st to 5th Grad-CAM images are, respectively, obtained from the 1st Block B, 2nd Block B, 3rd Block B, 5th Block B, and last Block A in Table S3.

We used the power of DL models to design a robust shoulder implant system that assists the orthopedic field, particularly shoulder arthroscopy. Manual identification of implants in X-ray scans requires highly experienced surgeons with plenty of time because minor errors cause somber consequences [9,12,13]. To address these problems, we propose an efficient DL-based classification ensemble network comprising our two designed CNNs (IFC-Net and MFC-Net) and a JMLP, as shown in Figure S2. We used the gradient-weighted class activation map (Grad-CAM) technique to illustrate the effectiveness of the two CNNs. Grad-CAM analyzes the gradient of the classification score with respect to the network-determined convolutional features and scores the significant region of the data [56]. In Figure 5 and Figure 6, we generated five Grad-CAMs of both CNNs for each class of the same input implant scan to guarantee a fair comparison. Figure 5 shows that IFC-Net appropriately learns the discriminative features of each class gradually. The visually discernible part of an implant lies in its head area, with a certain shape and number of holes. As shown in the rightmost column of Figure 5, IFC-Net successfully locates the discriminative part of the implants for all classes. In addition, Figure 6 shows that MFC-Net gradually learns the discriminative features of each class. The rightmost column in Figure 6 shows the visual class-specific regions obtained using MFC-Net. Furthermore, Figure 6 demonstrates that the class-specific regions, determined by MFC-Net, deviate from the visually discriminated regions and mingled with nondiscriminated regions. For example, the classes Cofield (first row) and Zimmer (last row) maximally deviate from the discriminated region, as compared to the other two classes (Depuy and Tornier). Therefore, the classification performance of the base model [27] of MFC-Net for C1 (Cofield class) and C4 (Zimmer class) was less than that of the base model [23] of IFC-Net for the same classes, which can be seen in the confusion matrix in Figure 4. However, Figure 6 shows that the class-specific region obtained by MFC-Net for the Tornier class (the third row) is larger in volume than that obtained by IFC-Net for the same class (the third row in Figure 5). Therefore, the classification performance of the base model [27] of MFC-Net for C3 (Tornier class) was 3.33% higher than that of the base model [23] of IFC-Net for the same class, which can be seen in the confusion matrix in Figure 4. Moreover, Figure 5 shows that the class-specific region obtained by IFC-Net for the Depuy class (the second row) is larger in volume than that obtained by MFC-Net (the second row in Figure 6). Therefore, the classification performance of the base model [27] of MFC-Net for C2 (Depuy class) was 2.57% less than that of the base model [23] of IFC-Net for the same class, which can be seen in the confusion matrix in Figure 4. 

Additionally, we analyzed the classification performance of our IMFC-Net class-wise and found that all classes have an AR higher than 80%, except for C1, as shown in Figure 4c. We analyzed the FN samples of C1 and inferred that C1 data samples were misclassified owing to the small size of C1 and high inter-class similarities with other classes. We completely examined the FN cases of C1, which were misclassified as C2 and C4 due to their structural similarities and dominant sizes of C2 and C4 over C1. Figures S5 and S6 show the high inter-class structural similarities of C1 implants with that of C2 and C4, respectively. Moreover, C2 is the largest class, having 71.77% more data samples than C1, whereas C4 is the second-largest class, with 44.3% more data samples than C1. Therefore, the classification performance of C1 was affected by C2 and C4. Although the data samples of all classes are augmented, an imbalanced distribution exists among the classes.

## 5. Conclusions

In this study, different DL-based frameworks (IFC-Net, MFC-Net, and IMFC-Net) have been proposed to identify different types of shoulder implants in X-ray scans. These frameworks automatically recognize different types of prostheses and assist the medical experts in fitting them to the human body and setting apparatus for personalized medicine. We compared the performance of the proposed models with that of the comparison models to demonstrate the outstanding performance of the proposed models. MFC-Net achieved 3.38% higher accuracy than that of the presented method in [19]. IFC-Net is efficient, with 31.72% fewer parameters than that of the presented method in [20] (i.e., 41.7 M [20] > 28.4 M (IFC-Net)) and 2.14% higher accuracy than that achieved in [20]. To further improve the efficiency of IFC-Net, we designed IMFC-Net, which was an ensemble of two subnetworks (IFC-Net and MFC-Net). IMFC-Net outperforms the model presented in [20] in terms of performance gain, with 4.01% higher ACC than that of [20], where it has 18.4% fewer parameters than that of the model presented in [20] (i.e., 41.7 M [20] > 34 M (Proposed)). Furthermore, IMFC-Net outperformed all state-of-the-art models, with the ACC, AP, AR, and F1 of 89.09%, 89.54%, 86.57%, and 87.94%, respectively. In addition, IMFC-Net ranked first, with considerable performance in experiments with and without augmentation. Computer-based diagnostic methods can enhance the surgeon’s performance and provide more robust solutions than subjective methods. More than 10% of implants are not recognized before revision arthroscopy, and this incapability leads to longer operation times, a need to replace more components, increased surgical complexity, higher healthcare expenses, more blood loss, and longer recovery time. The proposed model is efficient and can minimize the revision complexities of implants. The experimental results highlight the outstanding performance of our models. Moreover, our model is publicly available. 

Despite the good performance of the proposed model, this study has a few limitations that can be addressed in future studies. First, the size of the employed dataset was limited. In the future, we will upgrade the dataset by including other types of implants, such as knees and hips, of different modalities. In addition, we will perform cross-dataset validation to design a comprehensive classification framework. Second, despite the augmentation of the dataset, the class imbalance problem still persists. Owing to the large size of data, the class imbalance in learning needs to be resolved. Moreover, we aim to design a general implant system based on diverse data to address real-world problems.

## Figures and Tables

**Table 1:** Significance of using the CP block in IFC-Net and MFC-Net for the shoulder implant dataset. (CP: convolution pooling, ACC: accuracy, AP: average precision, AR: average recall, F1: F1.score, Std: standard deviation, unit: %).

Model	Performance without CP Block				Performance with CP Block			
	ACC ± Std	AP ± Std	AR ± Std	F1 ± Std	ACC ± Std	AP ± Std	AR ± Std	F1 ± Std
Inception-V3 [23] for IFC-Net	85.77 ± 4.55	86.25 ± 5.14	81.76 ± 4.76	83.85 ± 3.93	87.22 ± 4.47	87.66 ± 5.39	84.32 ± 4.15	85.88 ± 3.98
MobileNet-V2 [27] for MFC-Net	83.22 ± 3.96	81.41 ± 4.38	79.8 ± 7.3	80.56 ± 5.7	83.86 ± 4.88	83.29 ± 4.98	80.54 ± 8.8	81.79 ± 6.67

**Table 2:** Performance comparison of the submodules with that of the proposed model using the shoulder implant dataset. (ACC: accuracy, AP: average precision, AR: average recall, F1: F1.score, Std: standard deviation, unit: %).

Model	ACC ± Std	AP ± Std	AR ± Std	F1 ± Std
MFC-Net	83.86 ± 4.88	83.29 ± 4.98	80.54 ± 8.8	81.79 ± 6.67
IFC-Net	87.22 ± 4.47	87.66 ± 5.39	84.32 ± 4.15	85.88 ± 3.98
Proposed (IMFC-Net)	89.09 ± 4.55	89.54 ± 3.82	86.57 ± 7.63	87.94 ± 5.49

**Table 3:** Performance analysis between the sequential and end-to-end training of the proposed model using the shoulder implant dataset as an ablation study. (ACC: accuracy, AP: average precision, AR: average recall, F1: F1.score, Std: standard deviation, unit: %).

Model	Training Method	ACC ± Std	AP ± Std	AR ± Std	F1 ± Std
Proposed (IMFC-Net)	End-to-End	86.56 ± 2.96	85.53 ± 4.09	84.07 ± 5.18	84.7 ± 3.78
	Sequential	89.09 ± 4.55	89.54 ± 3.82	86.57 ± 7.63	87.94 ± 5.49

**Table 4:** Comparative performance analysis of the average results of ten-fold cross-validation between the state-of-the-art and proposed models using rotational invariant augmentation (RIA) [20] for the shoulder implant dataset. (ACC: accuracy, AP: average precision, AR: average recall, F1: F1.score, Std: standard deviation, unit: %).

Model	ACC ± Std	AP ± Std	AR ± Std	F1 ± Std
VGG-16 [19,20,22]	68.72 ± 7.34	66.39 ± 8.94	66.51 ± 9.18	66.32 ± 8.47
VGG-19 [19,20,22]	65.82 ± 5.96	63.99 ± 6.16	62.76 ± 6.61	63.29 ± 5.83
DarkNet-53 [32]	53.13 ± 6.02	44.47 ± 4.94	40.71 ± 5.08	42.4 ± 4.72
NASNet [19,20,33]	80.48 ± 5.22	78.76 ± 6.28	76.83 ± 7.73	77.68 ± 6.41
ResNet-18 [20,24,35]	77.38 ± 8	77.01 ± 9.42	73.6 ± 9.35	75.13 ± 8.75
ResNet-50 [19,20,24]	80.27 ± 6.53	79.71 ± 7.62	76.61 ± 6.76	78.08 ± 6.83
ResNet-101 [24]	82.18 ± 5.39	82.43 ± 7.85	77.98 ± 9.29	79.92 ± 7.53
DenseNet-201 [19,20,34]	84.24 ± 2.45	84.57 ± 3.66	82.1 ± 4.52	83.28 ± 3.7
Inception-V3 [23]	85.77 ± 4.55	86.25 ± 5.15	81.76 ± 4.76	83.85 ± 3.93
MobileNet-V2 [27]	83.22 ± 3.96	81.41 ± 4.38	79.81 ± 7.3	80.56 ± 5.70
DRE-Net [20]	85.08 ± 3.12	84.75 ± 4.54	83.61 ± 4.3	84.15 ± 4.09
Proposed (IMFC-Net)	89.09 ± 4.55	89.54 ± 3.82	86.57 ± 7.63	87.94 ± 5.49

## Data Availability

Not applicable.

## Supplementary Materials

The following are available online at https://www.mdpi.com/article/10.3390/jpm12010109/s1, Figure S1: Images showing low inter-class and high intra-class variabilities, Figure S2: Architecture of the proposed framework (IMFC-Net), Figure S3: Architecture of the CP block, Figure S4: Graphs of accuracies and losses for training and validation verifies the convergence of the three proposed networks without overfitting, Figure S5: Structural similarities between C1 (Cofield class) and C2 (Depuy class), Figure S6: Structural similarities between C1 (Cofield class) and C4 (Zimmer class), Table S1: Tabular description of rotational invariant augmentation (RIA) [20] training, validation, and testing data of ten-fold cross-validation (unit: images), Table S2: Detailed layer-wise architecture of the proposed IFC-Net, Table S3: Detail layer-wise architecture of the proposed MFC-Net, Table S4: Detail layer-wise architecture of JMLP, Table S5: A comparison between the state-of-the-art methods for dental and hip implant identification in X-ray scans, and Table S6: A comparison between the state-of-the-art methods and our method for knee and shoulder implant identification in X-ray scans.
