# Supplementary material for: Artificial Intelligence-Based Solution in Personalized Computer-Aided Arthroscopy of Shoulder Prostheses
Source: J Pers Med. 2022 Jan 14;12(1):109. doi: 10.3390/jpm12010109 (PMC8780458; doi:10.3390/jpm12010109)
Supplement: Supplementary file 1 [file jpm-12-00109-s001.zip › jpm-1512936-supplementary.pdf]

Supplementary Materials

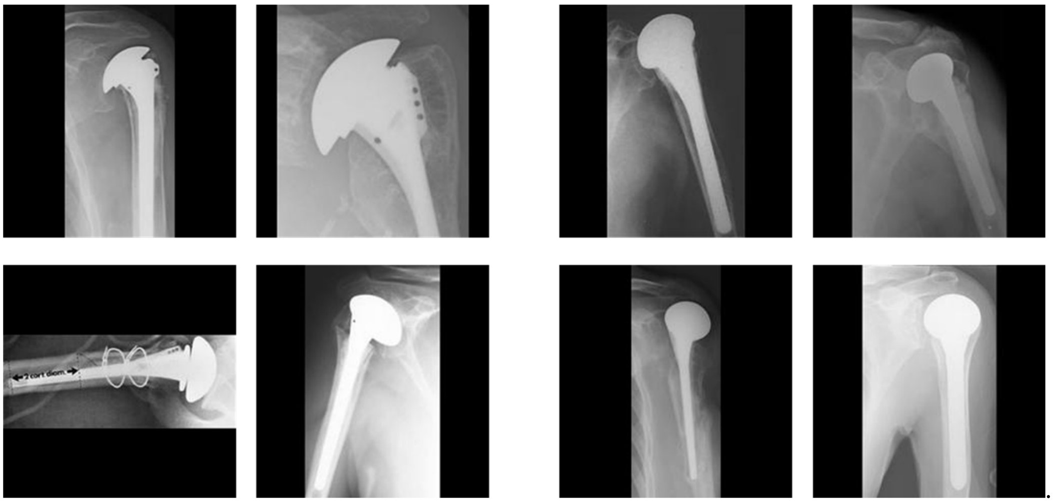

(a) **(b)**

Figure S1. Images showing low inter-class and high intra-class variabilities: **(a)** images of the Depuy manufacturer showing a high intra-class variance, and **(b)** low inter-class variabilities. In **(b)**, the cases of four manufacturers: Zimmer, Tornier, Depuy, and Cofield are shown in the top left, top right, bottom left, and bottom right images, respectively.

Table S1. Tabular description of rotational invariant augmentation (RIA) [20] training, validation, and testing data for ten-fold cross-validation (unit: images).

Fold #	RIA-Training	Validation	Testing
1	19,906	12	47
2	19,832	12	49
3	19,906	12	47
4	19,869	12	48
5	19,943	12	46
6	19,832	13	48
7	19,869	12	48
8	19,906	12	47
9	19,832	12	49
10	19,906	12	47

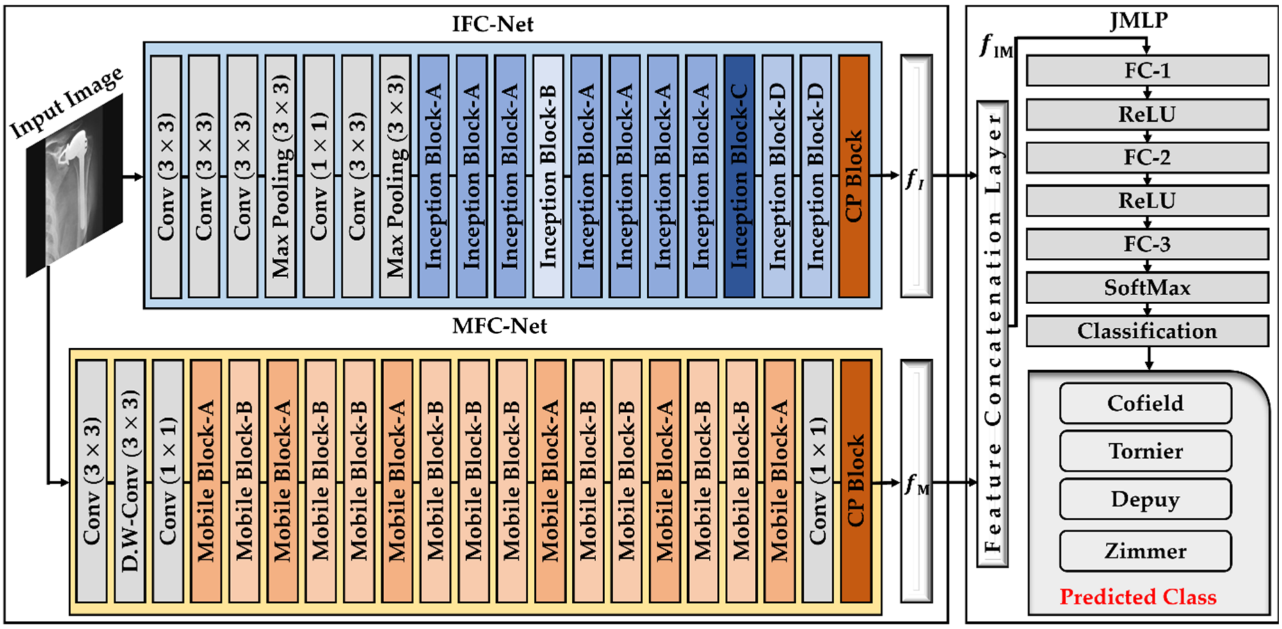

Figure S2. Architecture of the proposed framework (IMFC-Net).

Table S2. Detailed layer-wise architecture of the proposed IFC-Net.

Layer Name	# Iterations	Input Size	Output Size	Filter Size	# Parameters
**Input**	**1**	**-**	299 × 299 × 3	-	0
Conv	3	299 × 299 × 3	147 × 147 × 64	3 × 3<br>3 × 3<br>3 × 3	28,896
Max Pooling	1	147 × 147 × 64	73 × 73 × 64	3 × 3	0
Conv	1	73 × 73 × 64	73 × 73 × 80	1 × 1	5,360
Conv	1	73 × 73 × 80	71 × 71 × 192	3 × 3	138,816
Max Pooling	1	71 × 71 × 192	35 × 35 × 192	3 × 3	0
Block A	3	35 × 35 × 192	35 × 35 × 288	1 × 1 1 × 1<br>1 × 1 5 × 5 3 × 3 1 × 1<br>3 × 3	819,984
Block B	1	35 × 35 × 288	17 × 17 × 768	1 × 1<br>3 × 3 3 × 3<br>3 × 3	1,153,920
Block C	4	17 × 17 × 768	17 × 17 × 768	1 × 1<br>7 × 1 1 × 1<br>1 × 1 1× 7 1 × 7 1 × 1<br>7 × 1 7× 1<br>1 × 7	6,713,172
Block D	1	17 × 17 × 768	8 × 8 × 1280	1 ×1 1 × 1<br>3 × 3 1 × 7<br>7 × 1	1,699,584
Block E	2	8 × 8 × 1280	8 × 8 × 2048	1 × 1 1 × 1<br>1 × 1 1 × 3 3 × 3 1 × 1<br>3 × 1 1 × 3<br>3 × 1	11,127,936
Block CP	1	8 × 8 × 2048	1 × 1 × 64	8 × 8<br>1 × 1	6,758,614
**Total number of parameters : 28,446,282**					

Table S3. Detail layer-wise architecture of the proposed MFC-Net.

Layer Name	# Iterations	Input Size	Output Size	Filter Size	# Parameters
Input	1	-	224 × 224 × 3	-	0
Conv-1	1	224 × 224 × 3	112 × 112 × 32	3 × 3	960
DW-Conv	1	112 × 112 × 32	112 × 112 × 32	3 × 3	384
Conv-2	1	112 × 112 × 32	112 × 112 × 16	1 × 1	560
Block A	1	112 × 112 × 16	56 × 56 × 24	1 × 1<br>3 × 3<br>1 × 1	5,352
Block B	1	56 × 56 × 24	56 × 56 × 24	1 × 1<br>3 × 3<br>1 × 1	9,144
Block A	1	56 × 56 × 24	28 × 28 × 32	1 × 1<br>3 × 3<br>1 × 1	10,320
Block B	2	28 × 28 × 32	28 × 28 × 32	1 × 1<br>3 × 3<br>1 × 1	30,528
Block A	1	28 × 28 × 32	14 × 14 × 64	1 × 1<br>3 × 3<br>1 × 1	21,504
Block B	3	14 × 14 × 64	14 × 14 × 64	1 × 1<br>3 × 3<br>1 × 1	165,312
Block A	1	14 × 14 × 64	14 × 14 × 96	1 × 1<br>3 × 3<br>1 × 1	67,488
Block B	2	14 × 14 × 96	14 × 14 × 96	1 × 1<br>3 × 3<br>1 × 1	239,040
Block A	1	14 × 14 × 96	7 × 7 × 160	1 × 1<br>3 × 3<br>1 × 1	156,576
Block B	2	7 × 7 × 160	7 × 7 × 160	1 × 1<br>3 × 3<br>1 × 1	644,160
Block A	1	7 × 7 × 160	7 × 7 × 320	1 × 1<br>3 × 3<br>1 × 1	476,160
Conv-3	1	7 × 7 × 320	7 × 7 × 1280	1 × 1	413,440
Block CP	1	7 × 7 × 1280	1 × 1 × 64	7 × 7<br>1 × 1	3,293,014
**Total number of parameters : 5,533,942**					

Table S4. Detail layer-wise architecture of JMLP.

Layer Name	# Iterations	Input Size	Output Size	Filter Size	# Parameters
Concat	1	1 × 1 × 64<br>1 × 1 × 64	1 × 1 × 128	-	0
FC-1	1	1 × 1 × 128	1 × 1 × 64	1 × 1	8,256
FC-2	1	1 × 1 × 64	1 × 1 × 64	1 × 1	4,160
FC-3	1	1 × 1 × 64	1 × 1 × 4	1 × 1	260
Softmax	1	1 × 1 × 4	1 × 1 × 4	-	0
Classification	1	1 × 1 × 4	4	-	0
**Total number of parameters: 12,676**					

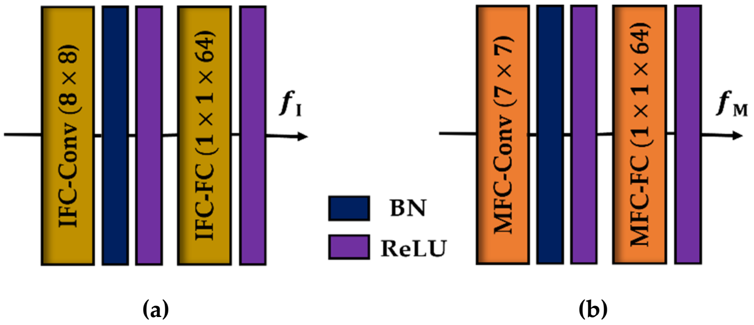

Figure S3. Architecture of the CP block: **(a)** CP block of IFC-Net, **(b)** CP block of MFC-Net. (BN: Batch normalization, ReLU: Rectified linear unit).

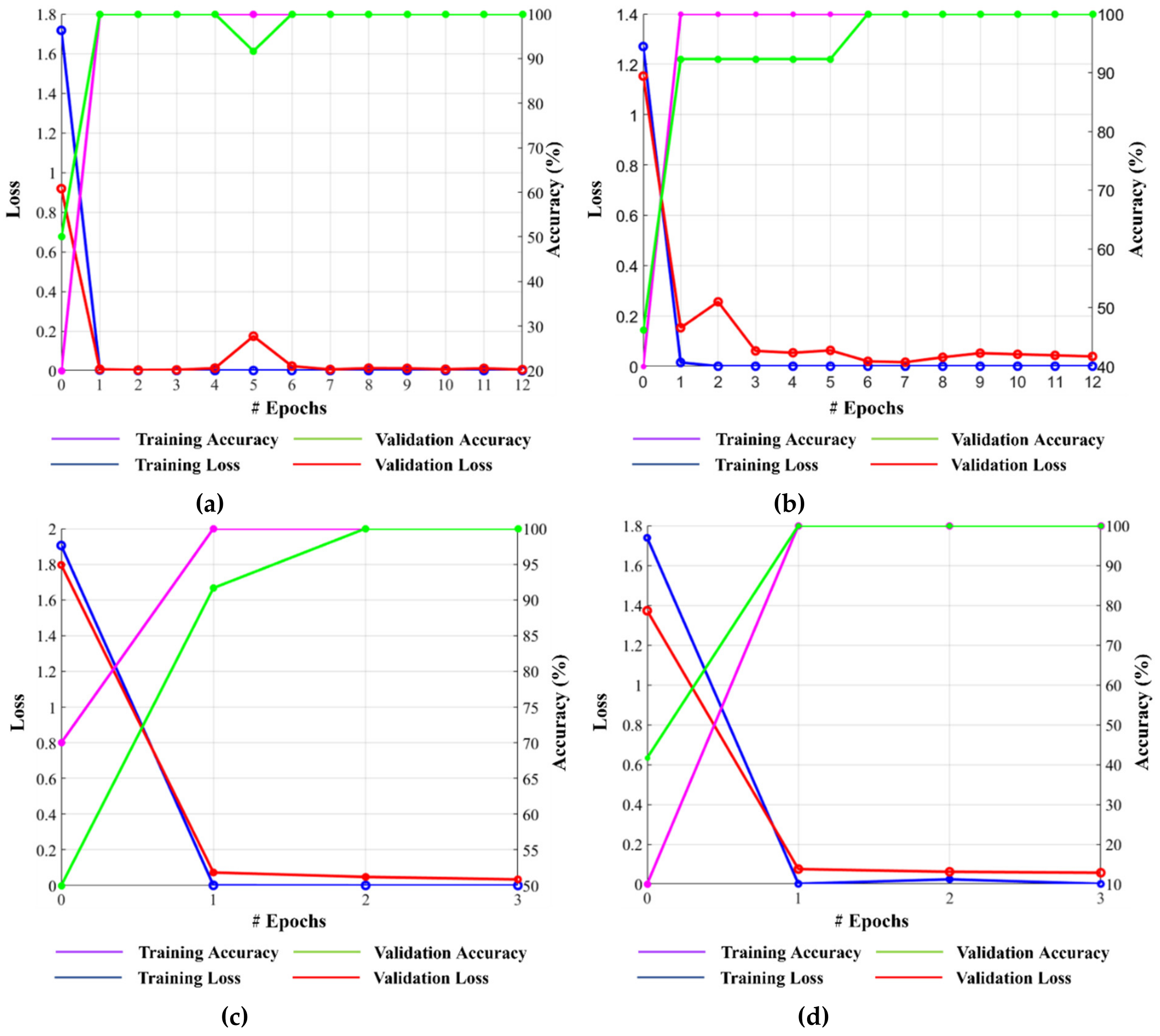

Figure S4. Graphs of accuracies and losses for training and validation verifies the convergence of the three proposed networks without overfitting: **(a)** IFC-Net, **(b)** MFC-Net, **(c)** IMFC-Net (sequential), and **(d)** IMFC-Net (end-to-end).

S1. Related Studies

Before the advent of DL strategies, conventional machine learning (ML) techniques were used to identify different types of implants. The ML techniques include different algorithms for classification, such as k-nearest neighbor (k-NN), support vector machine, Naïve Bayes, and logistic regression. Moreover, the application of ML techniques has revolutionized the orthopedic field. The accuracy of implant placement has been improved using intraoperative computer-assisted navigation and patient-specific equipment. After the successful application of the DL models in object detection, classification, and localization, various DL algorithms have been successfully used to design classification [36,37] and segmentation frameworks [38–40] to diagnose different diseases. In addition, DL can be employed in orthopedic surgery. However, the use and potential advantages of DL-based models in arthroplasty are limited. Therefore, we listed the existing studies on the classification of different types of implants using the DL and ML techniques.

S1.1. Classification of the Dental Implants

Dental implants have been classified using handcrafted feature-based methods. For example, in [41], a simple image processing technique, called active contour, was used for the segmentation of dental implants in the 2D X-ray images. Furthermore, 91% of implants were accurately classified using the k-NN algorithm.

Five deep convolutional neural networks (CNNs) were used in [42] to classify 11 types of dental implants in X-ray images. Variants of the pre-trained visual geometry group (VGG) [22] models were used by fine-tuning and achieving an accuracy of 93.5%. However, the images were manually cropped and not validated by an expert. In [43], the performance of the DL algorithms was compared with that of dental professionals on the classification of six types of dental implants. High classification results of 95.4% area under the curve (AUC) were achieved, whereas the DL network outperformed dental professionals. However, they did not make a comparison with state-of-the-art CNNs in addition to not using a validation dataset. In [44], variants of residual networks were employed for the classification of 12 different models of dental implants. Multi- and single-task models were designed for the classification of the dental implant and implant treatment stages, respectively. The multi-task model outperformed other models by achieving an accuracy of 99.72%. A pre-trained Inception-V3 was used in [45] to identify three different types of dental implants. The transfer learning technique and fine-tuned Inception-V3 were used to achieve an AUC of 97.1%. However, their study lacks a comparative study with the existing state-of-the-art networks. Five different deep CNNs were used in [46] to classify four types of dental implants in X-ray images. All networks achieved an accuracy of more than 90% with a small dataset.

S1.2. Classification of the Hip Implants

An artificial intelligence (AI)-based system was designed to identify hip arthroplasty models in the postoperative anteroposterior (AP) X-rays [47]. In [47], YOLO-V3 [32] was used for the stem detection of hip implants and categorizing them using a six-layer CNN. The dataset distribution was highly disproportionate to low-quality images. Similarly, an AI-based classification framework was proposed in [48] to recognize the AP arthroplasty implants from 18 different manufacturers. An accuracy of 99.6% was achieved by training the network for up to 1,000 epochs. The power of DL algorithms was used in [49] for the classification of three types of hip arthroplasty implants on plain radiographs. High classification accuracy of 100% was achieved for a small dataset using online augmentation. The transfer learning approach with the DenseNet-201 [34] model was used to identify nine different implant models for total hip replacement [50]. However, the dataset was not uniformly distributed, and the model exhibited a modest performance for the minority classes.

S1.3. Classification of the Knee Implants

Various types of knee implants have been classified using different ML techniques. In [51], the template matching technique, Sobel operator, and binarization for segmenting the knee implants were used in the X-ray scans, which yielded an accuracy of 90% for frontal X-ray images. However, the model was unable to obtain a high accuracy for lateral X-ray images.

A DL system was proposed in [52] for classifying three datasets of knee implants. Total knee arthroplasty (TKA) and unicompartmental knee arthroplasty were classified using variants of the pre-trained residual network. However, only two models of TKA were used, which limited the generalizability of the network and had the possibility of overfitting. In [53], knee implants were classified into nine different models from four manufacturers using a DL algorithm. High classification accuracy of 99% was achieved by training the model for up to 1,000 epochs. The images were preprocessed by cropping them manually. Furthermore, only AP radiographs were used, and the model was not trained on the lateral radiographs. In [54], a pre-trained residual CNN was employed, and seven different types of knee implant models were classified. The images were cropped manually, and the training data were augmented to achieve an accuracy of 100%. A DL network based on a dense block was proposed in [55] to identify four different types of knee implants. The dilated convolution was employed, which resulted in high classification accuracy for five-fold cross-validation using a pre-trained CNN. In addition, the ablation studies using normal 2D-convolution and state-of-the-art pre-trained networks were not considered.

S1.4. Classification of the Shoulder Implants

Handcrafted feature-based techniques have been used to classify the shoulder implants supplied by various manufacturers. In [18], the histogram equalization and Hough transform were applied to detect the shoulder implants based on their head circles. The images were preprocessed using different filters, including bilateral and median blur.

Moreover, few DL-based studies have been conducted to recognize the shoulder implants based on manufacturers. A DL system was proposed in [35] for the binary classification of shoulder implants. TSA and RTSA were classified using a pre-trained residual network based on the transfer learning techniques. Five types of TSA implant models were classified using a separate classifier for each model. An implant dataset was collected from online archives. Therefore, the authenticity of the label was questioned. In [19], the first DL-based study was presented for the classification of the shoulder prostheses supplied by four different manufacturers. The non-DL and DL algorithms were compared in addition to a comparison between the pre-trained and non-pre-trained DL models. Ten-fold trials were performed using various pre-trained CNNs, which yielded a maximum accuracy of 80%. However, the validation dataset was not used, and the experiments were limited to a closed-world scenario. In [20], a DL-based ensemble network was proposed for the robust classification of different shoulder prostheses. The proposed network in [20] outperformed the method presented in [19] by achieving an accuracy of 85.92%. However, their ensemble model was replete with many parameters, and the state-of-the-art methods were not validated using a validation dataset. For a fair comparison, we used a validation dataset to validate all state-of-the-art methods, including our proposed networks. Our networks, IMFC-Net and IFC-Net, outperform the networks presented in [19,20] in terms of accuracy. With the performance gain, the number of parameters of IMFC-Net is 18.4% less than that presented in [20], indicating higher efficiency.

Tables S6 and S7 compares the advantages and limitations of the previous study for recognizing different types of implants on radiographs.

Table S5. A comparison between the state-of-the-art methods for dental and hip implant identification in X-ray scans. ML: Machine learning, DL: Deep learning, ACC: Accuracy, F1: F1.score, AP: Average precision, AR: Average recall, SPEC: Specificity, SEN: Sensitivity.

**Implant**	**Dental**						Hip			
**Technique**	**ML**	**DL**					DL			
**Author**	**Morais et al. [41]**	**Sukegawa et al. [42]**	**Lee et al. [43]**	Sukegawa et al. [44]	Lee et al. [45]	Kim et al. [46]	Kang et al. [47]	Karnuta et al. [48]	Borjali et al. [49]	Borjali et al. [50]
**# Classes**	11	11	6	12	3	4	29	18	3	9
**Model**	k-NN	VGG	Deep CNN	ResNet	Inception-V3	MobileNet-V2	YOLO-V3	Inception-V3	DenseNet-201	DenseNet-201
**Result (%)**	91% of implants are detected	AR: 90.7, AP: 92.8, ACC: 93.5, F1: 91.6	AUC: 95.4, SEN: 95.5, SPEC: 85.3	ACC:99.08, AR: 98.86	AUC: 97.1	ACC: 97	AUC: 99	ACC: 99.6, SEN: 94.3, SPEC: 99.8	ACC: 100	ACC: 100 for five of nine designs
**Strength**	Used a simple image processing technique and an ML method.	Analyzed the effects of a small dataset with four variants of the VGG-network.	The network outperformed the dental professionals.	The model classified implant brands and treatment phases at the same time.	The network exhibited an acceptable performance for periapical and panoramic images.	Computationally less expensive.	High AUC for a large number of classes.	High classification performance.	Robust recognition.	The model takes less time as compared to professionals.
**Limitation**	An automatic approach can be used for segmentation.	Images are manually cropped and the VGG network can be replaced with deep state-of-the-art networks.	They did not use a validation dataset and did not make a comparison with the state-of-the-art CNNs.	The validation dataset was not used, and experiments were not performed for the open-world configuration.	Ten times augmenting training data caused computational complexity.	Images containing more than one implant are manually segmented.	Preprocessing was needed, and labeling was performed manually by a non-expert.	The model required more training time for 1000 epochs.	Computationally expensive.	The model exhibited a moderate performance for minority classes.

Table S6. A comparison between the state-of-the-art methods and our method for knee and shoulder implant identification in X-ray scans. ML: Machine learning, DL: Deep learning, ACC: Accuracy, F1: F1.score, AP: Average precision, AR: Average recall, SPEC: Specificity, SEN: Sensitivity.

**Implant**	Knee					Shoulder				
**Technique**	ML	DL				ML	DL			
**Author**	Bredow et al. [51]	Yi et al. [52]	Karnuta et al. [53]	Belete et al. [54]	Yan et al. [55]	Stark et al. [18]	Yi et al. [35]	Urban et al. [19]	Sultan et al. [20]	**Proposed**
**# Classes**	1	2	9	7	4	4	2	4	4	4
**Model**	Template matching	ResNet	Inception-V3	ResNet	Deep TKA classifier	Hough transform	ResNet	NASNet	DRE-Net	IMFC-Net
**Result (%)**	ACC: 70-90	AUC: 100	ACC: 99, AUC: 99, SPEC: 99	ACC: 100	AP: 97, AR: 97, F1: 97	AP: 77, F1: 64	AUC: 97, SEN: 95, SPEC: 90	ACC: 80.4, AP: 80, AR: 75, F1: 76	ACC: 85.92, AP: 85.33, AR: 84.11, F1: 84.69	ACC: 89.09, AP: 89.54, AR: 86.57, F1: 87.94
**Advantages**	They used simple techniques with less memory consumption and time complexity.	Training time is less than 60 minutes, and testing time is less than 2 seconds.	High classification performance.	They used a validation dataset and achieved perfect results.	They used the power of dilated convolution.	An automatic classification framework based on simple conventional image processing techniques.	High performance to classify TSA and RTSA.	- Shows the significance of non-DL models over DL models.<br>- Shows the significance of pre-trained CNNs over non-pre-trained CNNs.	An ensemble model with high classification performance.	- An efficient ensemble model with fewer parameters and higher classification performance.<br>- Validation dataset is used.
**Limitation**	The model is unable to get high accuracy for lateral X-ray images.	The model lacks generalizability due to a small number of classes.	Model requires more training time for 1000 epochs.	Manual segmentation can be replaced with automated segmentation.	Pre-trained CNNs can perform better than non-pre-trained CNNs.	Preprocessing is needed.	Augmentation is needed and the model classifies the multiclass problem as a binary class.	- Validation dataset is not used.<br>- Results can be optimized.	- An ensemble model with many parameters<br>- Computationally complex.	Requires more training time.

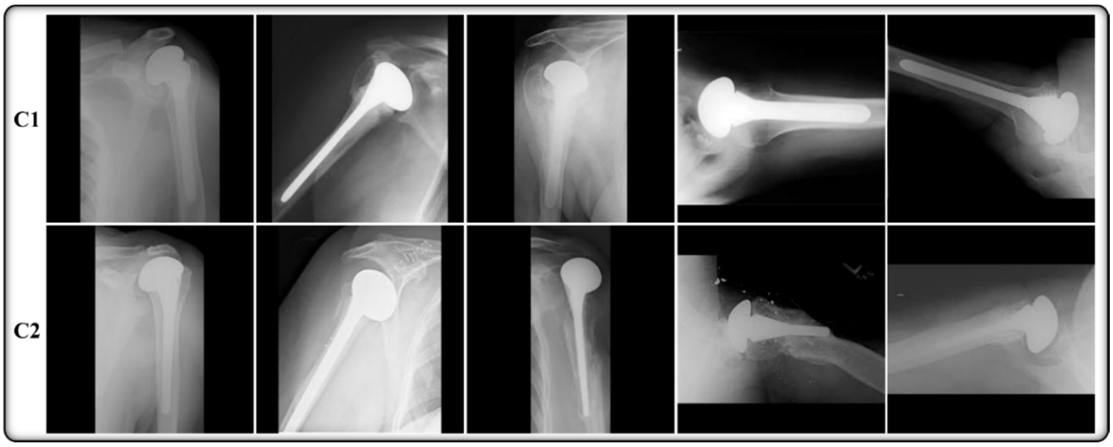

Figure S5. Structural similarities between C1 (Cofield class) and C2 (Depuy class).

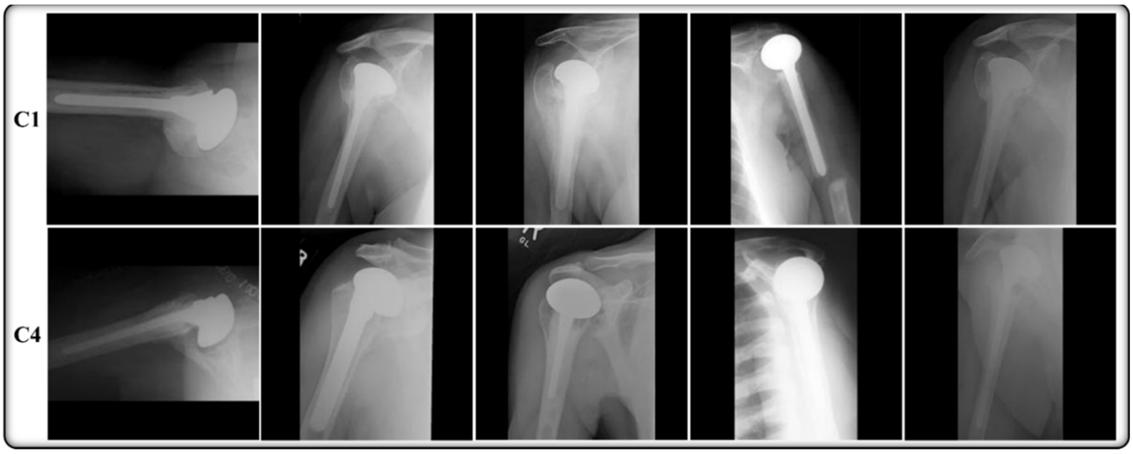

Figure S6. Structural similarities between C1 (Cofield class) and C4 (Zimmer class).
